# Non-Steroidal Anti-Inflammatory Drugs Are Inhibitors of the Intestinal Proton-Coupled Amino Acid Transporter (PAT1): Ibuprofen and Diclofenac Are Non-Translocated Inhibitors

**DOI:** 10.3390/pharmaceutics17010049

**Published:** 2025-01-02

**Authors:** Carsten Uhd Nielsen, Sebastian Jakobsen, Maria L. Pedersen

**Affiliations:** Department of Physics, Chemistry and Pharmacy, University of Southern Denmark, Campusvej 55, DK-5230 Odense, Denmark; seja@sdu.dk (S.J.); mariap@sdu.dk (M.L.P.)

**Keywords:** hPAT1, human proton-coupled amino acid transporter, NSAIDs, ibuprofen, inhibitor, Caco-2, TEVC, AlphaFold, molecular modeling

## Abstract

**Background/Objectives**: The proton-coupled amino acid transporter (PAT1) is an intestinal absorptive solute carrier responsible for the oral bioavailability of some GABA-mimetic drug substances such as vigabatrin and gaboxadol. In the present work, we investigate if non-steroidal anti-inflammatory drug substances (NSAIDs) interact with substrate transport via human (h)PAT1. **Methods**: The transport of substrates via hPAT1 was investigated in Caco-2 cells using radiolabeled substrate uptake and in *X. laevis* oocytes injected with *hPAT1 cRNA*, measuring induced currents using the two-electrode voltage clamp technique. The molecular interaction between NSAIDs and hPAT1 was investigated using an AlphaFold2 model and molecular docking. **Results:** NSAIDs such as ibuprofen, diclofenac, and flurbiprofen inhibited proline uptake via hPAT1, with IC_50_ values of 954 (logIC_50_ 2.98 ± 0.1) µM, 272 (logIC_50_ 2.43 ± 0.1) µM, and 280 (logIC_50_ 2.45 ± 0.1) µM, respectively. Ibuprofen acted as a non-competitive inhibitor of hPAT1-mediated proline transport. In hPAT1-expressing oocytes, ibuprofen and diclofenac did not induce inward currents, and inhibited inward currents caused by proline. Molecular modeling pointed to a binding mode involving an allosteric site. **Conclusions:** NSAIDs interact with hPAT1 as non-translocated non-competitive inhibitors, and molecular modeling points to a binding mode involving an allosteric site distinct from the substrate binding site. The present findings could be used as a starting point for developing specific hPAT1 inhibitors.

## 1. Introduction

**Figure 1:**
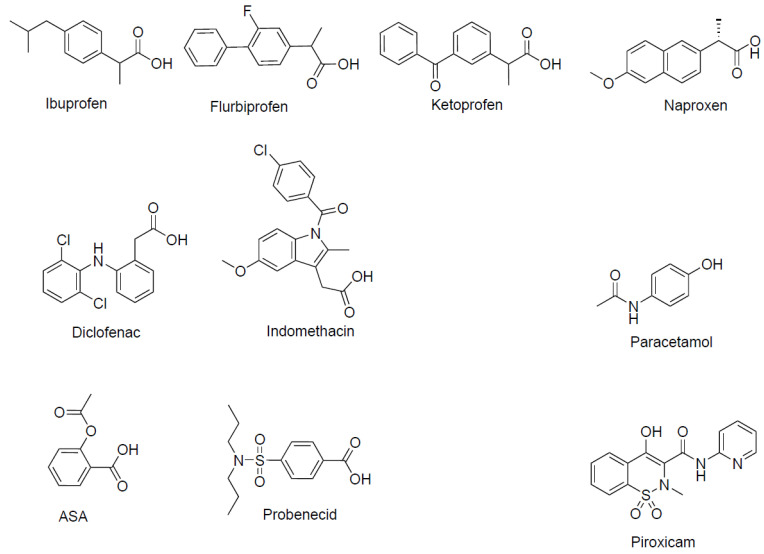
Chemical structure and names of the compounds investigated for hPAT1 interaction. ASA: Acetylsalicylic acid.

Non-steroidal anti-inflammatory drug substances (NSAIDs, Figure 1) are commonly used to treat mild pain and fever [1]. Interestingly, NSAIDs such as diclofenac and ibuprofen interact with several membrane transport proteins of the solute carrier (SLC) family found in the small intestine, such as hPEPT1 [2], MCT1 [3], and SMCT1 [4,5], where especially hPEPT1 is well recognized for its role as a drug transporter of valine-based acyclovir prodrugs, tripeptide-mimetic β-lactam antibiotics, and other dipeptide mimetics such as bestatin and δ-aminolevulinic acid [6]. Moreover, NSAIDs have been found to interact with members of the ATP-binding cassette (ABC) transporter family such as BCRP [7]; P-gp [8,9]; and MRP1, 2, and 4 [10,11,12,13]. In other tissues of the body, e.g., in the cells of the kidney, NSAIDs such as ibuprofen, diclofenac, and flurbiprofen have been shown to be inhibitors of OAT1 without being transported themselves [14]. Likewise, other members of the SLC22 family, such as OAT2, OAT3, and OAT4, have been shown to be inhibited by NSAIDs [15]. Collectively, ibuprofen inhibits several solute carriers and transporters, which do not share substrates or much amino acid sequence similarity. The pattern of broad transporter recognition of, e.g., ibuprofen could indicate that ibuprofen is exerting adverse effects on the membrane by altering the structural or physical properties of the membrane, resulting in unspecific apparent inhibition of the transporter. However, some studies have shown that in membranes with cholesterol, water transport is not altered by ibuprofen [16,17], whereas water and structural effects are changed in membranes without cholesterol [16], suggesting that ibuprofen in native membranes is not causing an unspecific membrane effect with related effects on membrane transporters. Two transporters from different SLC families that do share a limited substrate overlap are the proton-coupled di/tri-peptide transporter PEPT1 (SLC15A1) and the proton-coupled amino acid transporter PAT1 (SLC36A1). They are both proton-coupled symporters, and despite the fact that the hPEPT1 amino acid (AA) sequence (708 AA) and hPAT1 sequence (476 AA) do not share any similarity, they share a small number of substrates; Gly-Sar, δ-aminolevulinic acid, and Gly-Gly are substrates of both solute carriers [18,19], while dipeptides such as Gly-X_aa_ are substrates for hPEPT1, but inhibit substrate transport via hPAT1 [20]. As ibuprofen is an inhibitor of hPEPT1 [2], we thus hypothesized that other NSAIDs, including ibuprofen and diclofenac, could also interact with hPAT1. Since ibuprofen and other NSAIDs inhibit different classes of transporters, investigating if NSAIDs interact with membrane transporter proteins has so far been carried out on a case-by-case basis. However, with the emergence of AlphaFold, it has become possible to predict the interactions between NSAIDs and membrane transporters with computational tools [21,22,23]. There is thus a need to establish which membrane transporters interact with NSAIDs to identify structural ligand–protein interactions, which are likely not competitive interactions. Such knowledge will help to find new allosteric binding sites and to pave the way for in vitro-based assessments of drug–drug interactions for drugs utilizing membrane transporters for absorption or elimination. Moreover, finding allosteric binding sites may help with the development of selective inhibitors. Since PAT1 is a transporter of drug substances such as vigabatrin, d-cycloserine, betaine, δ-aminolevulinic acid, and gaboxadol (for an overview, see [24]), this is a further reason to investigate if ibuprofen could interact with PAT1. Hence, the aim of the present study was to investigate if NSAIDs such as diclofenac and ibuprofen interact with PAT1. The compounds studied, NSAIDs along with paracetamol and probenecid, are shown in Figure 1. The ability of the compounds shown in Figure 1 to inhibit PAT1-mediated proline transport in vitro was investigated in Caco-2 cells, known to express hPAT1 in the apical membrane [25], and based on the results, ibuprofen and diclofenac were further investigated for their ability to act as substrates or non-translocated inhibitors in hPAT1-expressing *Xenopus Laevis* oocytes. Moreover, the molecular interaction between NSAIDs and hPAT1 was investigated using an AlphaFold2 model and molecular docking.

## 2. Materials and Methods

### 2.1. Materials

Caco-2 cells were obtained from Deutsche Sammlung von Mikroorganismen und Zellkulturen (DSMZ) (Leibniz Institute, Braunschweig, Germany). Transwell inserts were obtained from Corning Life Sciences (New York City, NY, USA), and all other cell culture plastic products from Sigma-Aldrich (Merck KGaA, Darmstadt, Germany). Dulbecco’s Modified Eagle’s Medium (DMEM), L-proline (Pro), L-glutamic acid (L-Glu), L-glutamine (Gln), non-essential amino acids (NEAA), 2-(4-morpholino)ethanosulfonic acid (MES), penicillin/streptomycin, 5-hydroxy-L-tryptophan (5-HTP), sodium chloride, potassium chloride, sodium bicarbonate, calcium nitrate, magnesium sulfate heptahydrate, calcium chloride dihydrate, and magnesium chloride were purchased from Sigma-Aldrich (Merck KGaA, Darmstadt, Germany). Bovine serum albumin (BSA), sodium bicarbonate solution 7.5%, fetal bovine serum (FBS), and Hank’s Balanced Salt Solution (HBSS) with calcium and magnesium without sodium bicarbonate and phenol red were purchased from Thermo Fisher Scientific (Waltham, MA, USA). 4-(2-hydroxyethyl)-1-piperazineethansulfonic acid (HEPES) and Triton X-100 were purchased from AppliChem GmbH (Darmstadt, Germany). Ethanol > 99.7% was purchased from VWR (Radnor, PA, USA), and ultrapure water was obtained from an *in-house* Water Purification systems Milli-Q Gradient, Millipore (Merck, Darmstadt, Germany). L-[2,3,4,5-^3^H]-proline (75 Ci mmol^−1^, radiochemical purity > 97%) and Ultima Gold scintillation liquid were obtained from Perkin Elmer (Waltham, MA, USA). All other chemicals were of reagent or laboratory grade, including the racemic NSAIDs.

### 2.2. Methods

#### 2.2.1. Cell Culture

Experiments using Caco-2 cells were performed as previously described [26]. For toxicity investigations, cells were incubated for 6 days in a 96-well plate and equilibrated with pH 7.4 at 37 °C on an orbital shaker (220 rpm) from Troemner (Thorofare, NJ, USA), and 150 µL of the test solutions were added. Briefly, 1% Triton X-100 was used as a positive control and considered as 100% LDH release, while the LDH release in the HBSS buffer was taken as baseline and 20 mM L-proline was used as a negative control. An amount of 100 µL of the sample solutions was withdrawn after 5 min, whereas Triton X-100 was incubated for 30 min and centrifuged before transferring the solutions containing Triton X-100. The LDH content was assayed according to the manufacturer’s instructions utilizing an LDH-kit (Cat. No. 04 744 926 001, Roche, Basal, Switzerland), where 100 µL of the reaction mixture was added to the wells and incubated for 30 min while protected from light. An amount of 50 µL of stop solution was added to the wells, followed by spectrophotometric determination (λ = 490 nm, Elisa reader; Bie & Berntsen, Herlev, Denmark).

#### 2.2.2. Uptake Experiments in Caco-2 Cells

Both the donor solutions and uptake studies in the cell experiments were performed as previously described [26]. The uptake of proline was investigated in HBSS buffer at either pH 6.0 or pH 7.4 for 5 min. All uptake experiments were performed using 1 μCi mL^−1^ radiolabeled ^3^H-Pro alone or in buffer solution supplemented with a non-labeled concentration of proline for concentration-dependent studies or NSAIDs for inhibition studies at either a single concentration or for affinity determinations. The concentration of the NSAIDs was chosen to be 2.5 mM, since the K_m_-values for the natural substrates are mostly in the range of 2–20 mM [24]. For some compounds, lower concentrations were used due to solubility limitation. The disintegration per minute (DPM) was measured using a liquid scintillation counter (LSC) (Tri-Carb 2900TR or TriCarb 4910TR, Perkin Elmer, Waltham, MA, USA).

#### 2.2.3. Xenopus Laevis Oocytes and Two-Electrode Voltage Clamp Measurements

*SLC36A1 cRNA* was synthesized through the in vitro transcription of linearized pGEM-HE-SLC36A1 as previously described by Frolund et al. [19]. Experiments on stage VI oocytes from *Xenopus laevis* frogs (collagenase-treated) were performed as previously described [26]. All experiments were performed on at least 4–6 oocytes per condition.

#### 2.2.4. AlphaFold2 Model and Molecular Docking

The predicted 3D structure of hPAT1 (SLC36A1) was downloaded from the AlphaFold database [21] (AF-Q7Z2H8-F1-v4, UniProt ID: Q7Z2H8) and loaded into Maestro (13.6), which is part of the Schrödinger Suite [27]. Maestro’s Protein Preparation was used to add missing hydrogens, assign bond orders, generate protonation states at pH = 7.4, and perform restrained minimization using the OPLS4 force field. The ligands (glycine, L-proline, and the studied NSAIDs) were prepared using LigPrep to generate 3D conformations and protonation states at pH = 7.0 ± 2.0. The orthosteric binding site was recognized as an occluded site near G62, T63, and G64, which forms the transmembrane domain 1 (TM1) GXG motif commonly found in the substrate binding site of LeuT-fold transporters [28]. The proposed allosteric site for the binding of the NSAIDs was recognized as an intracellular cavity next to the orthosteric site. Glide was used to generate receptor grids and perform SP (standard precision) docking using default settings.

#### 2.2.5. Data Analysis

The total uptake of proline at pH 6.0 in Caco-2 cells was described by Michaelis–Menten-like kinetics:

(1) $$V_{o}=\frac{V_{max}\cdot \left[S\right]}{K_{m}+\left[S\right]}$$

where V_o_ is the initial uptake rate of or proline at a given proline concentration [S] in mM in the donor solution, V_max_ is the maximal uptake rate, and K_m_ is the Michaelis constant.

The inhibition of the total uptake of proline at pH 6.0 in Caco-2 cells was described by

(2) $$V_{o}=Bottom+\frac{Top-Bottom}{1+\frac{\left[I\right]}{{IC}_{50}}}$$

where V_o_ is the initial uptake rate of proline (in %) in the presence of various concentrations of ibuprofen, diclofenac, or flurbiprofen [I], Top is the maximal uptake rate of proline in the absence of inhibition (100%), and bottom is the uptake rate (%) at the maximal degree of inhibition.

#### 2.2.6. Statistical Analysis

Statistical analysis was performed in GraphPad Prism version 10.4. The obtained data were analyzed for statistical differences using a parametric one-way ANOVA test followed by Dunnett’s multiple comparison test. The following level of significance was used: *p* < 0.05 (*). The values are expressed as mean ± SEM. The data were obtained in at least 3 independent cell passages (n = 3) or at least 6 individual oocytes (n = 6).

## 3. Results

### 3.1. Various NSAIDs Inhibit PAT1-Mediated Uptake of Proline in Caco-2 Cells

**Figure 2:**
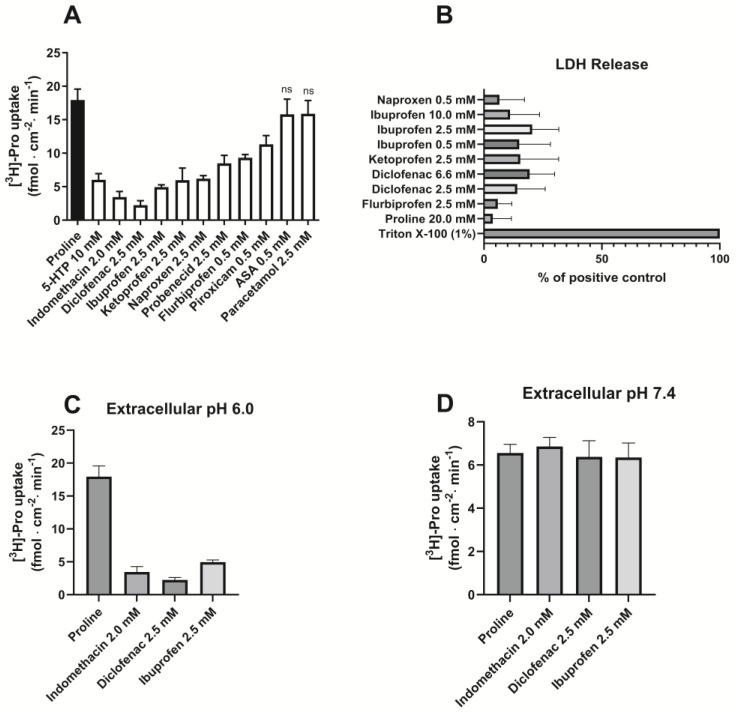
Proline uptake in Caco-2 cells measured in the absence or presence of NSAIDs. (**A**). The uptake rate of 13.3 nM [^3^H]-proline (1.0 µCi ml^−1^) in 10 mM MES HBSS buffer, pH 6.0 (control), and the presence of various NSAIDs and paracetamol (n = 3–4). (**B**). LDH release (n = 3). Each value represents the mean ± SEM. (**C**). The uptake rate of 13.3 nM [^3^H]-proline (1.0 µCi mL^−1^) in 10 mM MES HBSS buffer, pH 6.0 (control) and the presence of indomethacin, diclofenac, and ibuprofen (n = 3). (**D**). The uptake rate of 13.3 nM [^3^H]-proline (1.0 µCi mL^−1^) in 10 mM HEPES HBSS buffer, pH 7.4 (control), and the presence of indomethacin, diclofenac, and ibuprofen (n = 3).

The uptake of proline was measured for 5 min at pH 6.0 and was significantly reduced by the presence of 10 mM 5-HTP. Likewise, the uptake of proline was decreased by the presence of 2.0 mM indomethacin; 2.5 mM of diclofenac, ibuprofen, ketoprofen, naproxen, and probenecid; and 0.5 mM flurbiprofen and piroxicam, whereas 0.5 mM ASA or 2.5 mM paracetamol did not influence the uptake of proline (Figure 2A). To verify that the observed effects were not due to a general effect on the cell membrane, the release of lactate dehydrogenase (LDH) was measured using 1% Triton X-100 as a positive control (Figure 2B). The different NSAIDs did not cause increased LDH release compared to the negative proline control, which was present in approx. an 8–10 times higher concentration than the NSAIDs. To further address if the observations were a specific effect on hPAT1 or unspecific effects on the cell membrane, the uptake of proline was investigated at two different extracellular pH values, and these are compared in Figure 2C,D. The uptake of proline is clearly pH-dependent, with an almost 3 times higher proline uptake at pH 6.0 (Figure 2C) than at pH 7.4 (Figure 2D), which, compared with the effect of 10 mM 5-HTP (Figure 2A), support that the proline uptake in Caco-2 cells is mediated by hPAT1. The ability of the NSAIDs to inhibit proline uptake is only present at an extracellular pH of 6.0, whereas at pH 7.4, proline uptake is not affected by indomethacin, diclofenac, or ibuprofen.

### 3.2. Ibuprofen, Diclofenac, and Flurbiprofen Concentration-Dependently Inhibit PAT1-Mediated Uptake of Proline

**Figure 3:**
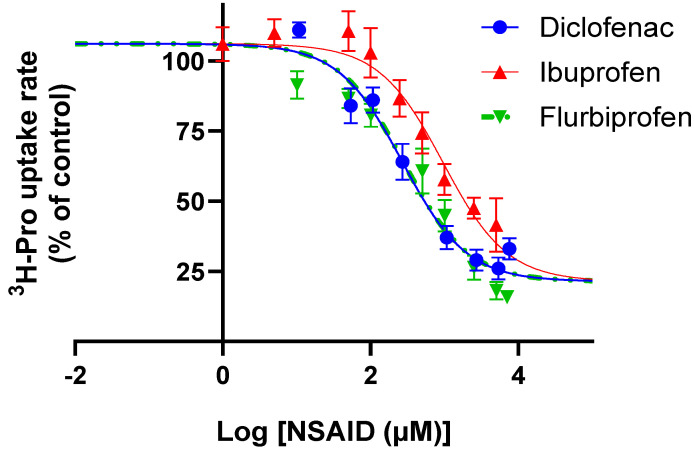
Concentrations-dependent inhibition of 13.3 nM [^3^H]-proline (1.0 µCi mL^−1^) uptake rate (%) at pH 6.0 in Caco-2 cells of diclofenac, flurbiprofen, or ibuprofen. The IC_50_ value was determined from Equation (2). Each value represents the mean ± SEM (n = 3).

The uptake of proline was investigated in Caco-2 cells in the presence of increasing concentrations of ibuprofen, diclofenac, and flurbiprofen (Figure 3). Under slightly acidic extracellular conditions, ibuprofen, diclofenac, and flurbiprofen inhibited hPAT1-mediated proline uptake, with IC_50_ values of 954 (logIC_50_ 2.98 ± 0.1) µM, 272 (logIC_50_ 2.43 ± 0.1) µM, and 280 (logIC_50_ 2.45 ± 0.1) µM, respectively (Figure 3). The experimental data were fitted to a common top and bottom value, which were 106 ± 2% and 21.4 ± 2.9%, respectively, with a Hill coefficient of 1.

**Figure 4:**
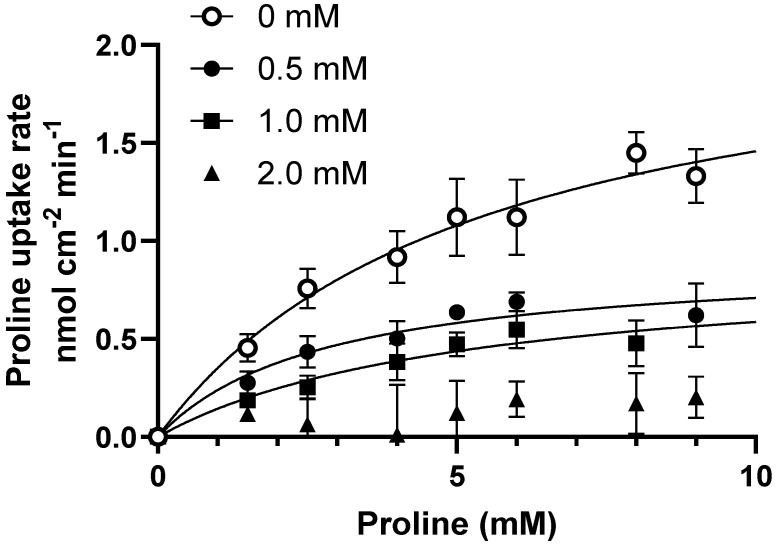
Concentration-dependent proline uptake in Caco-2 cells in the absence or presence of ibuprofen at pH 6.0. The uptake rate of 13.3 nM [^3^H]-proline (1.0 µCi mL^−1^) with 0–10 mM L-proline (o), in the presence of 0.5 mM ibuprofen (●), 1.0 mM ibuprofen (■), or 2.0 mM ibuprofen (▲). Each value represents the mean ± SEM (n = 3). The solid lines show the fit of the resulting data to Equation (1).

To investigate the underlying mechanism behind the inhibition of proline uptake, ibuprofen was further investigated. The concentration-dependent uptake of proline was measured in Caco-2 cells under slightly acidic conditions in the presence of different ibuprofen concentrations (Figure 4). The uptake of proline was saturable and in the presence of ibuprofen, and the K_m_-value remained statistically similar, while the V_max_ value decreased significantly with increasing ibuprofen concentrations (Figure 4 and Table 1). This inhibition pattern suggests a non-competitive interaction between hPAT1-mediated proline transport and ibuprofen.

### 3.3. Ibuprofen and Diclofenac Inhibit Proline-Induced Currents in PAT1-Expressing Oocytes

**Figure 5:**
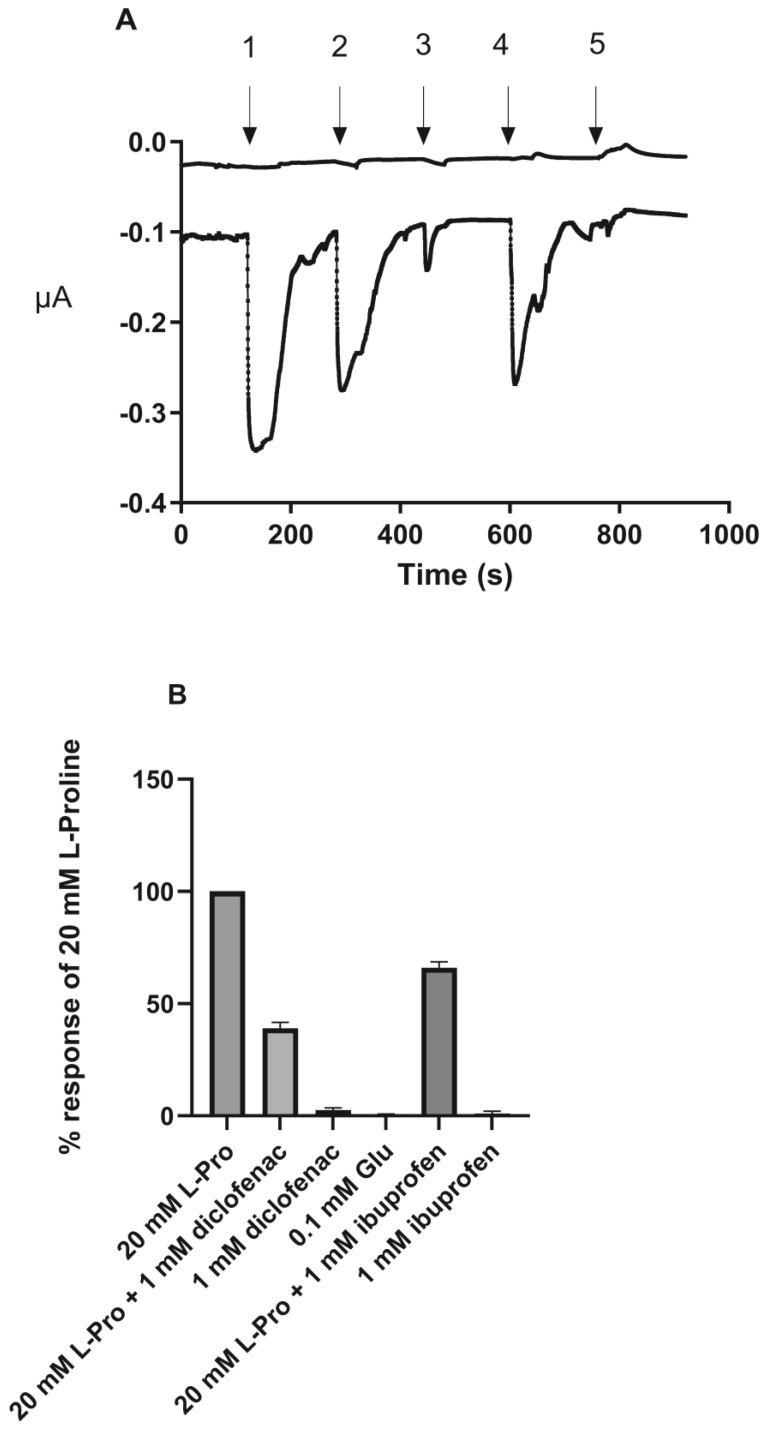
Two-electrode voltage clamp (TEVC) measurements in PAT1-expressing *Xenopus Laevis* oocytes. (**A**). The traces are representative of experiments performed on 4–6 different oocytes. The upper trace is a trace in a water-injected oocyte, whereas the lower trace is in a oocyte injected with *SLC36A1 cRNA*. The induced current in PAT1-expressing oocytes at a holding potential of −60 mV and continuously perfused with Ringer’s solution pH 6.0. 1: 20.0 mM L-Pro; 2: 20 mM L-Pro with 1.0 mM ibuprofen; 3: 1.0 mM ibuprofen; 4: 20.0 mM L-Pro with 1.0 mM diclofenac; 5: 1.0 mM diclofenac (**B**). Effect on the response induced by 20.0 mM proline by treatments 2–5 and 0.1 mM L-Glu as a control of a negatively charged amino acid. Each value represents the mean ± SEM (n = 4–6).

To investigate the effect of ibuprofen and diclofenac on PAT1-mediated transport in PAT1-expressing oocytes, TEVC recordings were conducted (Figure 5). Proline induced inward-directed currents in PAT1-expressing oocytes, whereas no change in baseline current was observed in water-injected oocytes (upper trace in Figure 5). The proline-induced signal was attenuated when ibuprofen or diclofenac was present in the proline-containing solution. Buffers containing ibuprofen or diclofenac alone did not induce an inward current, showing that they were not substrates of hPAT1 but acted as non-translocated inhibitors of hPAT1-mediated proline transport.

### 3.4. Proposed Binding Sites of Amino Acids and NSAIDs in the AlphaFold2 Model of hPAT1

**Figure 6:**
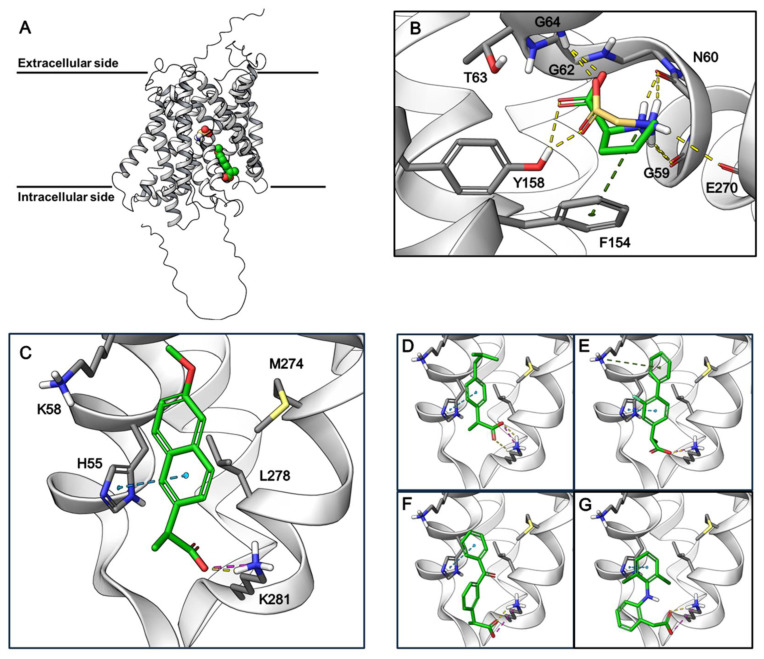
AlphaFold2-predicted structure of hPAT1 and docking poses of amino acids and NSAIDs. (**A**). AlphaFold2 structure of hPAT1 showing the proposed binding sites of glycine (in yellow) and naproxen (in green). (**B**). Docking poses of glycine (yellow) and L-proline (green) in the proposed orthosteric binding site of hPAT1. Yellow dashed lines: hydrogen bonds; green dashed lines: pi-cation interactions. (**C**–**G**). Docking poses of naproxen (**C**), ibuprofen (**D**), flurbiprofen (**E**), ketoprofen (**F**), and diclofenac (**G**) in the proposed allosteric binding site of hPAT1. Ibuprofen, flurbiprofen, and ketoprofen are racemic mixtures, and the stereoisomers with the best docking scores are depicted. Yellow dashed lines: hydrogen bonds; green dashed lines: π-cation interactions; blue dashed lines: π-π stacking; pink dashed lines: salt bridges.

To study the possible NSAID binding site in PAT1, the Alphafold2 (AF2) structure of hPAT1 was investigated since no experimental structure is available. PAT1 is part of the Amino acid-Polyamine-organoCation (APC) superfamily and is thus predicted to be organized into the LeuT-fold [29]. The hPAT1 AF2 model forms 11 transmembrane (TM) domains and appears to adopt an occluded inward-facing conformation (Figure 6A). The proposed orthosteric substrate binding site is recognized as an occluded pocket near the unwound regions of TM1 and TM6, with TM1 containing the classic GXG motif found in LeuT-fold substrate binding sites [28]. Using molecular docking, only the small natural substrates glycine and L-proline can fit in this small pocket, whereas the NSAIDs are unable to dock due to their size. The amino acids interact with PAT1 through hydrogen bonds to the backbone of TM1 and TM6 along with a π-cation interaction with Phe154 (Figure 6B). The binding pocket appears to be closed off to the intracellular side by Gln350, opposite of which a larger cavity exists that is connected to the cytosol. This cavity contains lysine and histidine residues, which we hypothesized could interact with the acidic groups and aromatic rings found in the NSAIDs. Docking to this cavity shows favorable interactions for the NSAIDS, where many of them are able to form salt bridges with Lys281 and π-π stacking interactions with His55 (Figure 6C–G). We thus hypothesized that this could be a potential allosteric binding site for some, if not all, of the NSAIDs.

## 4. Discussion

In the present study, chemically different NSAIDs along with paracetamol and probenecid were investigated for their ability to inhibit amino acid transport via the proton-coupled amino acid transporter, hPAT1, in human intestinal Caco-2 cells. All the investigated compounds except acetylsalicylic acid (ASA) and paracetamol inhibited the hPAT1-mediated cellular uptake of proline under slightly acidic conditions. As shown in Figure 1, ibuprofen, flurbiprofen, ketoprofen, and naproxen belong to the group of propionic acid derivatives, diclofenac and indomethacin are acetic acid derivatives, ASA belongs to the acetylated salicylates, piroxicam belongs to the enolic acids, paracetamol is not an NSAID, and probenecid is a sulfonamide used as a uricosuric drug, being a member of the benzoic acids. All the compounds, except paracetamol, are predominantly negatively charged at pH 6.0 due to the presence of a free carboxylic acid group, and for piroxicam, an acidic hydroxy group. Overall, the presence of a free negative charge appears to be the major determinant for binding to hPAT1. Yet, a few interesting deviations are that ASA, despite the negative charge, does not inhibit uptake, while probenecid does, with both having the carboxylic group attached directly to an aromatic ring. The remaining part of the molecule is either meta (ASA) or para (probenecid) to the carboxylic acid, which could suggest that a substituent in the meta position is not able to fit the binding area on the hPAT1 protein or that the concentration of ASA used is simply too low. The compounds with inhibitory properties have one negative charge at pH 6.0, whereas the hPAT1 natural amino acid substrates have a negatively charged carboxylic acid and a positively charged nitrogen, α-, β-, or γ-, to the carboxylic acid, which have been shown to be almost mandatory structural elements for binding to hPAT1 (see overview in [24]). This points to the fact that their binding occurs in an area in the protein distinct from the normal substrate binding sites. Supporting this hypothesis is the fact that ibuprofen inhibits proline uptake in a non-competitive manner, ibuprofen and diclofenac are not translocated by hPAT1, as shown by the TEVC recordings, and that the NSAIDs are unable to bind to the proposed orthosteric substrate binding site of the AF2 hPAT1 model. While the validity of the AF2 hPAT1 model has yet to be confirmed by experimental structures, it shows promise by capturing common features of the LeuT-fold substrate binding sites, such as the TM1 and TM6 unwound regions and the GXG motif [28], which actively participates in the molecular interactions with glycine and L-proline. Furthermore, the small substrate binding pocket is also in agreement with the natural substrates of hPAT1, which are primarily small amino acids such as glycine, proline, and alanine [24,25]. The non-translocated (and presumably competitive inhibitor due to the fact that tryptophan is competitive) and competitive hPAT1 inhibitor 5-hydroxytryptophan (5-HTP) [30] could not fit the substrate binding site when performing molecular docking. So, while 5-HTP might competitively bind the same substrate binding site in a different conformation, given its amino acid features, its size might prohibit it from undergoing a full translocation cycle, due to the size limitation of the occluded substrate pocket.

In terms of identifying a potent inhibitor of hPAT1, it seems promising that the NSAIDs can bind to an allosteric site of the protein and that their affinity is better than those of the natural substrates, which are mostly in the range of 2–20 mM [24]. A potential drawback could be that the NSAIDs also inhibit several other membrane transport proteins, and hence, the selectivity profile needs to be kept in mind at the early stage of developing new hPAT1 inhibitors. The proposed allosteric site in the hPAT1 AF2 model highlights molecular interactions that fit the common acidic and aromatic features of the NSAIDs. However, it should be noted that this site only seems accessible from the intracellular side, meaning that a given inhibitor must cross the plasma membrane before being able to bind. This seems likely to occur since it has been shown that ibuprofen transport across membranes was caused by passive permeation of the neutral form due to the pH-partitioning theory [31]. His55 is shown to interact with the NSAIDs in the proposed allosteric site of hPAT1, and this residue has been shown to be conserved throughout the SLC36 transporters and appears to be essential for their catalytic activity [32]. It is hypothesized that His55 is involved with the translocation of H^+^, further corroborating how an inhibitor might inhibit translocation by binding to this residue. Still, this proposed NSAID binding site has yet to be validated by other means. This could, e.g., be achieved through mutational studies that investigate whether the involved residues are needed for the inhibitory effect of the NSAIDs. With such validation, this allosteric site could potentially be used in virtual screening campaigns to look for other potent inhibitors of hPAT1. Amino acid transporters like hPAT1 have become popular drug targets in diseases, such as cancer, and the limited selection of hPAT1 inhibitors makes this a novel target, with the potential for therapeutic discoveries. The present study is thus a starting point for finding new inhibitors of hPAT1, yet the allosteric binding site must be validated before a rational design strategy can be undertaken.

Considering potential drug–drug interactions at the level of intestinal absorption, neither the FDA guidance on in vitro drug–drug interactions nor the EMA guidelines ([33,34]) provide any guidance for assigning hPAT1-mediated drug–drug interactions. Finding inspiration in the guidance for P-gp and BCRP, the intestinal concentration of the inhibitor divided by the IC_50_ value should be ≥10 to warrant a possible drug–drug interaction [33]. The intestinal concentration of ibuprofen can be predicted to initially 3.9–11.6 mM with a dose of 200–600 mg dissolved in 250 mL water, and similarly, the initial intestinal diclofenac concentration would be 0.68 mM with a 50 mg dose. The FDA ratio is then calculated to 4–12 for ibuprofen and 2.5 for diclofenac. In certain dose regimens within rheumatological diseases, ibuprofen is given for up to 2400 mg/day, divided into 3–4 doses for 4–6 weeks (recommendation in Denmark), while doses up to 3200 mg have been reported as the maximal safe daily dose [35]. Given these high doses and the FDA ratio of 12 at a 600 mg/dose, which is higher than 10, ibuprofen could mediate drug–drug interactions at the level of intestinal absorption of hPAT1 substrates. However, to our knowledge, no pharmacokinetic studies addressing such an interaction between, e.g., ibuprofen or gaboxadol, vigabatrin, or δ-aminolevulinic acid are available in the literature. Moreover, in terms of drug-nutrients interactions, it would be relevant to consider if the absorption of amino acids such as proline and glycine is affected in rheumatological patients taking high daily ibuprofen doses, and if this results in altered plasma amino acids levels. However, the intestinal surface area and the peristaltic movement in the intestine make it difficult to saturate drug transporters. In dogs, very high doses of L-tryptophan are needed to decrease the absorption of gaboxadol, and even when its absorption is affected, it results in decreased maximal plasma concentrations with increased t_max_, yet the overall absorption assessed as total AUC is not affected by the inhibitor [36]. Thus, the risk for clinically relevant drug–drug and drug–nutrient interactions through NSAIDs and PAT1 remains to be shown in an experimental or clinical setting.

## 5. Conclusions

NSAIDs interact with PAT1 as non-translocated non-competitive inhibitors, and molecular modeling points to a binding mode involving an allosteric site distinct from the substrate binding site. The present findings could be used as a starting point for developing specific PAT1 inhibitors.

## Figures and Tables

**Table 1:** Kinetic parameters for uptake of proline in the presence ibuprofen. * is significantly different from the V_max_ obtained in the absence of ibuprofen. The transporter-mediated intrinsic clearance (CL) was calculated as V_max_/K_m_.

Ibuprofen	K_m, app_ (mM)	V_max, app_ (nmol cm^−2^ min ^−1^)	Intrinsic CL (µL min^−1^ pr. cm^2^)
+0.0 mM	5.4 ± 2.2	2.2 ± 0.4	0.4
+0.5 mM	3.9 ± 1.9	0.9± 0.2 *	0.2
+1.0 mM	5.2 ± 3.7	0.9 ± 0.3 *	0.2
+2.0 mM	ND	ND	ND

## Data Availability

The original contributions presented in this study are included in the article. Further inquiries can be directed to the corresponding author.
